# Aberrant Dyspepsias

**Published:** 1908-12-12

**Authors:** Leonard Williams

**Affiliations:** Physician to the French Hospital, Assistant Physician to the Metropolitan Hospital


### Hospital Clinics.

### ABERRANT DYSPEPSIAS.

By LEONARD WILLIAMS, M.D., M.R.C.P., Physician to the French Hospital, Assistant Physician to the Metropolitan Hospital.

Among the commonplaces of medical practice the person who complains of dyspepsia under one of its numerous synonyms is surely the most common. This frequency of occurrence, with the opportunities it affords for observation, would lead one to suppose that the condition in question is one whose treatment is almost a matter of rule of thumb. That the very reverse is the fact, that of all the complaints for which we are consulted dyspepsia remains one of those which is least amenable to routine treatment, must be my excuse for once more reverting to so well worn a subject.

I have elsewhere* discussed at considerable length the question of functional dyspepsias; dyspepsias, that is, which are due to errors of secretion and motility, and I have shown that for all practical purposes they may be divided into two classes, namely, sthenic dyspepsia, caused by a superabundance of hydrochloric acid in the gastric juice; and the opposite condition of asthenic dyspepsia, in which all the elements of the gastric secretion, but notably this acid, are deficient in quantity. The differential diagnosis between these two conditions is generally easy, and even if we do make a mistake, the results of treatment will very soon proclaim the error. For, to impose upon a person who is already suffering from hyperacidity, an extra dose of his arch enemy is to ensure his speedy return with the indignant reproach that the medicine has increased his sufferings tenfold; and, in the same way, to apply alkalies to an organ whose secretions are already insufficiently acid is materially to intensify its difficulties. Such occurrences are rare, but they do occur, even when there seems to be no reasonable doubt as to which of the two types is present. The only certain way to avoid mistakes is the expedient of a test meal and the stomach-tube. I may be old-fashioned, but in a slight matter of this kind, speaking both as a physician and a patient, I would rather run the risk of a mistake between acid and alkali than face this humiliating ordeal.

In a general way I find that when there is a difficulty in coming to a conclusion as to whether the case in question is one of sthenic dsyepepsia or its opposite, it eventually turns out to be one which cannot, properly speaking, be placed in either category, but is due to some underlying cause which must be discovered and removed before either acid or alkali will have the desired effect. A large percentage of these aberrant cases are the result of causes which, for the want of a better term, we must call nervous or neurotic. Common instances are afforded by men on the Stock Exchange who lead strenuous, and even exhausting lives, who are exposed to periods of depression, varied by sudden volcanic explosions of excitement and panic, in whom the philosophic calm so necessary to good digestion is hardly ever obtainable except at a foreign health-resort, where telephones cease from troubling and "markets" are at rest. An instance drawn from another, though scarcely less familiar, sphere is presented by a young lady who consulted me some weeks ago, with all the signs of a sthenic dyspepsia, with this notable point of dissimilarity from the typical picture, that her acid symptoms began to trouble her as soon as the food obtained access to her stomach. The ordinary prescriptions of bismuth, soda and hydrocyanic acid, in combination with laxatives, produced no result, and so at her third visit I got her mother out of the room and demanded to know the nature of the silent sorrow which I felt certain she was nourishing. It soon emerged in the shape of a secret engagement which, should it leak out, would set the whole family by the ears. The combination of some bromide of potassium with a little worldly-wise advice cured that dyspepsia in a few days.

One of the most common causes of aberrant dyspepsias is that which, for some extraordinary reason, is the one which is most commonly overlooked. So common is it, indeed, that one feels almost ashamed to mention it. I mean dental caries. The teaching of the schools, and I say this without any implied reflection, tends to the too exclusive cultivation of the obscure in diagnosis and the heroic in treatment, with the sad result that the obvious and common-sensical become overlooked. Thus it happens that patients are suspected to be suffering from cancer, gastric ulcer, œsophageal stricture, hepatic, pancreatic and even splenic disease, when a few visits to a competent dentist will cause the disappearance of all their symptoms. We talk glibly of the gastro-intestinal toxins and their nefarious consequences, but we appear to think of them as lurking brigand-like in the inaccessible *rugæ* of the small intestine, when their real habitat is the commonplace cave of a decaying molar. That "washing in Jordan" should never be a popular proceeding with patients is comprehensible, because patients are generally in an epic mood; but why doctors should avoid it as a prescription is less obvious. That the avoidance frequently impairs professional credit is a matter of common experience.

Another condition which is very closely associated with intractable dyspepsias is nasal obstruction. No one can pretend that a nasal obstruction due to adenoids is now in danger of being overlooked. The very reverse is indeed the case, for adenoids are diagnosed, and even operated upon, in cases when they do not and never have existed. But that is by the way. Nasal obstruction may be due to causes other than adenoids, and such obstruction is a very common provoker or maintainer of a dyspepsia

* "Minor Maladies." 2nd Ed., p. 52. (Bailliere, Tindall, and Cox.)

which fails to conform to either of the two regular types, and remains obstinate to treatment by their appropriate remedies. Such was the case with a man whom I have known for many years; energetic, hard-working, capable, who at unequal intervals suffered from attacks of what both he and I agreed to call gouty dyspepsia. It was distinctly of the sthenic type, and the worst discomforts connected with it scarcely ever failed to yield to bismuth and soda. Nevertheless, even when taking the medicine he was seldom entirely free from flatulence, eructations, heart-burn and constipation. The enemy was always on his flank, to fall upon him unmercifully should he commit any dietetic indiscretion, or in the event of any extra pressure of work, and on the occasion of any mental anxiety. Matters continued thus for several years, until he was married. Not long after that event he came to see me with one of the usual attacks, and told me incidentally that his wife complained that he not infrequently snored, and that, in connection with this complaint on her part, he had himself noticed that he always awoke with his mouth open. I then, for the first time, tested his nasal airway, and found that it was practically blocked on the right side by a combination of spurs and a deviated septum. Since this condition was relieved, now over two years ago, he has never had any return of his trouble; or, if he has, it has been so slight in degree as to be readily amenable to ordinary treatment. This may seem an inconclusive story, but both he and I are quite convinced that the cause of his former troubles was the obstruction in his nose, an opinion which on my part is very strongly supported by other cases of a similar kind.

But if nasal obstruction is a common cause of obstinate digestive troubles, an even commoner is to be found in uncorrected errors of refraction. These errors give rise to eye-strain, and eye-strain in its turn provokes disturbances which are by no means limited to the eyes or their neighbourhood. The teaching of too many of the schools is to the effect that unless a person with a slight error of refraction complains of definite symptoms, then it is a work of supererogation to correct it. Such advice might be sound if all the symptoms of eye-strain were easily recognisable as such. But they are not. A person who is the subject of eye-strain may suffer from symptoms which neither he himself nor the vast majority of doctors would dream of referring to his vision.* It is beginning to be recognised, perhaps, that headaches, supra-orbital and other local neuralgias may be caused by visual defects, but it is seldom even admitted that dyspeptic troubles and many obscure and indefinable but very persistent miseries, which are either carelessly or ignorantly labelled neurotic, neurasthenic, or hysterical, may be due to the same cause. This attitude is not altogether surprising when we remember that in order to produce these results it is essential that the defect should be slight in degree; should be one, that is, which the patient himself, by contracting his ciliary muscle, can adequately correct. The grosser errors do not cause these symptoms, for the reason that no amount of ciliary contraction being sufficient to correct them, no effort is ever made. In the lesser degrees the effort, being successful, is not only made, but is maintained during the whole of the waking hours. It is this maintenance of muscular effort which is the crux of the whole situation, for the ceaseless and illegitimate contraction of the ciliary muscle means an equally ceaseless and illegitimate expenditure of nervous energy; the "electric power" intended for the motors in the various organs is all monopolised by the visual. There seems nothing to determine which of these organs will be the first to cry out that it is being starved of its due amount of nervous energy, and much of the trouble arises from the fact that its cry is almost invariably misunderstood and misinterpreted. In the case of the stomach the responsibility is generally placed upon the diet, which is pared and whittled both in quantity and quality until the fare of King Nebuchadnezzar may seem generous in comparison; while the organ itself is now soothed with papaveric caresses, and anon chastised with chilian scorpions, in the vain hope that it may thus be induced to make bricks without straw. For, unless the nervous energy or the motive power, or whatever else it may be termed, is prevented from leaking out through the crevice of that minor refractive error, the stomach will be deprived of its due share of this energy; with the result that symptoms in very sooth, though symptoms of an aberrant and baffling type, will continue to afflict the unfortunate possessor of the organ in spite of acid and alkali, and in spite, too, of their all too common and ridiculous association in the same mixture.

So impressed have I been during the last ten years with this aspect of obstinate dyspepsias that I now never fail to satisfy myself, at any rate in the case of a town dweller, and more especially in the case of a town dweller of over forty years of age, that an error of refraction is not at least a contributory cause in a case of troublesome indigestion which resists the ordinary remedies. If it is true, as I believe it to be, that the dentist cures more cases of indigestion than the physician, it is equally true that in the same direction the refractionist is more potent than the therapist.

A great many dyspepsias which are confidently assigned to the rubbish heap labelled "neurotic" are due to vaso-motor disturbances, and may thus be held to justify the label. The disturbance may take the form of an undue vaso-dilatation, leading to a subnormal blood pressure, or to the opposite condition of undue vaso-constriction, causing a supernormal blood pressure. It may, of course, be the result of faulty distributions of pressure for which errors of vascular tone are not primarily or even mainly responsible, as in the case of mitral disease. It is scarcely necessary to refer to such cases, because the person who fails to examine the heart in a case of dyspepsia will fail to examine it in a case of chorea, and is diagnostically past praying for. It is the vascular disturbances which own no such obvious cause which give rise to difficulties. In the case of undue general vaso-dilatation the

* *See* Ernest Clarke, "The Medical Aspect of Eye-strain," *Clin. Journal*, October 4, 1905.

*modus operandi* is not difficult to follow. The patient is, so to speak, living under the constant influence of nitrite of amyl; his peripheral arteries are relaxed, and there is thus less blood available for the work of the internal organs. Consequently the appeal for more blood for digestive work on the part of the stomach is very inadequately responded to, and symptoms arise whose severity is in direct ratio with the degree of general vaso-dilatation. A dyspepsia which is due to this state of matters may always be relieved by causing the patient to assume the recumbent posture immediately after a meal, but it can only be cured by removing the cause of the general vaso-dilatation. Of such causes there are many, but it is here only possible to refer to one of them. The condition, which is called pre-tuberculosis, is invariably characterised by an unduly low blood pressure, and we find in consequence that dyspepsia is very common in that condition. The aphorism that a chlorosis which does not yield to iron and aloes is probably tuberculous has been repeated to such good purpose that it is now well remembered; it would be a good thing if it were equally well remembered that a dyspepsia in a young person which yields not to acid or alkali and remains obstinate to bromides, is so suspicious of the pre-tuberculous state that confirmatory evidence should be carefully sought. The nature and relative value of such evidence I have already described and examined in detail.*

The opposite condition of unduly high blood-pressure frequently, if not invariably, carries a gastric disturbance of some kind in its train. The causation of high blood-pressure, in some at any rate of its aspects, is still a matter of speculation, but there seems no escape from the conclusion that it may be, and frequently is, due to endogenous toxins. These toxins would seem, in the majority of cases, to act slowly, so slowly that the existence of the high pressure is not even suspected until it has left its inexorable mark upon the arteries in some important organ, whose resulting degeneration has produced the symptoms from which the patient seeks relief. Here, then, is another, and by no means the least weighty, of the possibilities which should engage our attention where we have an aberrant dyspepsia to deal with. The use of the manometer is becoming more general every day, and such cases will therefore be overlooked with decreasing frequency, to the credit of the profession and to the satisfaction of the patients.

There is much archaic and pernicious nonsense still spoken about the superiority of the finger in estimating blood-pressures, which comes mainly from those who have never subjected the estimates thus obtained to the crucial test of the mercurial column; but ignorant prejudices proverbially die hard, and there is perhaps little to cavil at in the progress which this one is making towards the oblivion which it so richly merits.

Of the dyspepsias which result from high blood-pressure the best instance is probably that which may be drawn from a consideration of what occurs at the menopause. The process of menstruation must be regarded as an excretory process, so that the commencement of the climacteric marks as a rule the commencement of a period of insufficient excretion. Add to this the consideration that the internal secretion of the ovary is believed, on sufficient grounds, to be both vaso-dilator and a toxin destroyer, and it is not surprising to find that at the "gloaming of life," as the French poetically call it (*l'âge crépusculaire*), the blood distribution becomes deranged. The derangement shows itself as an elevation which is always definite and is not infrequently sufficiently alarming to warrant very active interference. For reasons into which it is impossible to enter here, this rise of pressure exercises a particularly unfavourable effect upon the vessels in the splanchnic area, and of these vessels it is, as one would expect, the gastric which show the greatest disturbance, with the result that dyspepsia, almost invariably of the sthenic type, is one of the commonest of the manifestations of the menopause. Any attempt to treat such a dyspepsia without very special attention to the state of the blood-pressure is to court certain failure, and in order to reduce that pressure we must bear in mind the above-mentioned factors in its causation.

The fact that an excretory organ has been lost and that its absence is not yet compensated for, will suggest gentle stimulation of the other emunctories, of which the skin is in this connection by no means the least important. The absence of the internal secretion may be met by giving ovarian extract by the mouth, a procedure which I believe to be of the greatest benefit. Ichthyol in five-grain pills is also useful, and is perhaps the best of all drugs for combating the vague subjective discomforts which are apt to appear at this time. When the blood-pressure is really high, *i.e.* over 200 mm. Hg., and the above means fail to reduce it, I never hesitate to recommend venesection. This little operation has in several cases within my knowledge been the means of a "miraculous" cure of very troublesome climacteric dyspepsias.

I feel that I must not leave this question without a reference to a form of dyspepsia which is associated with the menopause, but which has none of the characters of that just noticed. The processes peculiar to the climacteric affect different women differently, but they seldom fail to produce an instability of the nervous system, which in extreme cases proceeds to definite insanity. Short of this, one of the forms which it assumes is an abnormal craving for sedatives, and if the craving is satisfied there is very apt to ensue an irritable condition of the stomach which gives rise to symptoms of indigestion. The sedative usually employed is, of course, alcohol, with regard to which it is necessary to remember that its excessive devotees fly to it, not as a stimulant, but as a narcotic, and that it is narcotic only when taken in doses large enough to act as an irritant on the gastric mucosa. There is a great deal of secret drinking at the time of the menopause, even among those who up to that period have been strictly temperate, so that the possibility of such a factor being at work in producing or maintaining a dyspepsia should not be forgotten.

*Op. cit.* p. 33.

